# Deliberative Processes by Health Technology Assessment Agencies: A Reflection on Legitimacy, Values and Patient and Public Involvement Comment on "Use of Evidence-informed Deliberative Processes by Health Technology Assessment Agencies Around the Globe"

**DOI:** 10.34172/ijhpm.2020.46

**Published:** 2020-04-07

**Authors:** Mireille Goetghebeur, Marjo Cellier

**Affiliations:** ^1^Department of Management, Evaluation and Health Policy, School of Public Health, University of Montreal, Montreal, QC, Canada.; ^2^Research Center, University Hospital Center Ste Justine, Montreal, QC, Canada.

**Keywords:** Decision-Making, Health Technology Assessment, Ethics, Patient and Public Involvement (PPI), Multicriteria Approaches

## Abstract

Legitimacy of deliberation processes leading to recommendations for public financing or clinical practice depends on the data considered, stakeholders involved and the process by which both of these are selected and organised. Oortwijn et al provides an interesting exploration of processes currently in place in health technology assessment (HTA) agencies. However, agencies are struggling with core issues central to their legitimacy that goes beyond the procedural exploration of Oortwijn et al, such as: how processes reflect the mission and values of the agencies? How they ensure that recommendations are fair and reasonable? Which role should be given to public and patient involvement? Do agencies have a positive impact on the healthcare system and the populations served? What are the drivers of their evolution? We concur with Culyer commentary on the need of learning from doing what works best and that a reflection is indeed needed to "enhance the fairness and legitimacy of HTA."

The article by Oortwijn et al provides important and interesting information on current processes in health technology assessment (HTA) agencies.^1^ This effort is laudable, as there are is a diversity of approaches used throughout the world to support the HTA process and the deliberation. The steps leading to deliberation, defined in their evidence-informed deliberative processes (EDP) framework provides a footprint to explore these processes, by focusing on the mechanisms in place, using public documents and a survey of the International Network of Agencies for Health Technology Assessment (INHATA) members with a 54% response rate. The authors conclude that critical elements for conducting HTA and reporting HTA are in place. However, respondents also indicated that guidance is needed for key aspects such as selecting technologies and criteria to be used for selecting those technologies to be appraised, the appraisal process per se, as well as for communicating decisions and their underlying arguments, and for appeal. This is in line with the report of Kristensen et al, stating that standards in HTA are somewhat blurry, in particular regarding deliberative processes.^2^ Deliberation is an essential aspect of democracy and when fully accomplished holds the promise of a transformation of evidence into knowledge and wisdom. The discussion below builds on the commentaries of Culyer in particular regarding his reflection on the need to learn by doing, changes needed to enhance HTA, the embedding of ethics in the HTA process, the authority of the exercise and the collective thinking it fosters.^3,4^

Although the EDP builds on the framework accountability for reasonableness (A4R),^5^ which was set forth more than 20 years ago to address procedural ethics, through four conditions that can enhance legitimacy,^6^ there is no mention in the Oortwijn paper of ethics, nor on the alignment of processes with the mission and values of agencies. However, HTA are currently struggling with core issues central to their legitimacy such as:

- How processes in place reflect the mission and values of the agencies?
- How these processes ensure that recommendations made are fair and reasonable? Which attributes of the deliberative process are essential to achieve such recommendations?
- Which role is given to the chief interested parties, the public and patients?
- Do agencies have a positive impact and create value for the populations served?
- What are the strengths, weaknesses, opportunities and threats of HTA?
- What are the drivers of their evolution?

An exploration of the core procedural and substantive values of eight HTA agencies from Europe and the Americas was performed previously through a survey and a focus group discussion^7^; this work revealed key values common to the participating agencies, including scientific rigor, transparency, independence, and stakeholders involvement.

Regarding scientific rigor, traditionally, agencies have focused on quantitative data, relying on the concept that objectivity provides the best answer to questions raised by HTA. However, could rigorous integration of qualitative research, including structured consultation of patients, citizens and relevant stakeholders, analyses of the socio-political and the organizational contexts, identification of ethical aspects across all the dimensions of evaluation, contribute to a better assessment? Culyer assumes that a good deliberative process tackles ethical issues.^3^ It can be argued though that not everybody has the capacity to infer ethical aspects and that pragmatic ethical data holds the promise of enlightening the collective thinking which takes place during the deliberation. What would be needed for HTA agencies to reflect on the deeper meaning of scientific rigor, and as a public servant, ethical rigor, and adjust their practices as needed?

Transparency combines both procedural and substantive aspects, including which methods were used, which data was selected, and which arguments – elaborated by whom – were used to generate recommendations for public coverage. This being directly linked to the value of scientific rigor, and whether or not quantitative data is sufficient for informed appraisal, collecting narrative data from various participants raises additional questions, such as: which participants were involved throughout the process; how and why were they selected; what were the conflicts of interests and how were they managed? With this in mind, what would be needed for HTA agencies to achieve these different levels of transparency?

Independence often refers to the independence from the Ministry of Health to which the HTA agency is making recommendations for public financing. However, the level of independence in the choice of the interventions to be appraised is also to be taken into account, as well as whether agencies are appraising a diversity of needed healthcare interventions. Do agencies appraise high intensity techno-centred interventions and low intensity people-centred innovations? Interventions developed by the milieu of care and interventions developed by the industry? Are the comparative interventions on which the deliberation is based adequately selected? Bearing in mind that HTA agencies would likely enhance their legitimacy by diversifying the interventions they appraise and the comparators they used as a basis for their recommendations, there is the question as to how and why they should do so.

Finally, regarding stakeholder engagement, HTA agencies tend to consult, at some point in the process, clinicians with expertise and experience pertaining to the intervention being appraised, sometimes with high levels of conflict of interest. To avoid these conflicts, should the agencies consult clinicians with a diversity of opinions on the intervention? Should thus patient and public consultations be also diversified? What would HTA agencies need to be able to diversify the stakeholders involved, and the nature of their involvement?

In the same vein, should deliberative committees include patients and citizens, to bring their interests and concerns to the table? This point is in line with the question raised by Culyer regarding the authority of the work. One can ponder where indeed lies the authority to resolve ethical dilemmas that may arise and to make fair and reasonable recommendations for public financing? Is it the authority of clinical expertise borne by clinicians, the authority of experiential expertise borne by patients, the authority of health system organisation and governance borne by managers, the authority of population welfare concerns borne by citizens, by ethicists? All of the above? Other necessary authorities? Can the analysts provide a committee with such diversity of background with relevant data (clinical, populational economical, organizational, socio-ethical) in a manner that can be understood by all to ensure full consideration of issues at stake? Can the deliberation chairs ensure that, as proposed by Habermas, “each participant has an equal opportunity to be heard, to introduce topics, to make a contribution, to suggest and criticize proposals?”^8^

Regarding the important point on the authoritative scope of HTA raised by Culyer, in many jurisdictions, HTA agencies recommendations are most often directly transformed into policy decisions; policy-makers tend to rely on the HTA deliberative committee arguments, including its pondering on social matters and health distributional issues. Articulating these concepts within the A4R and making sure they are tackled at some point are critical in a context of rapidly increasing inequalities, in the midst of a rise of an often technocentric rather than human-oriented care, being brought forth by innovations such as telemedicine, precision medicine, artificial intelligence, and regenerative medicine.^9,10^

The A4R has been criticized for providing limited guidance on the substantive aspects of the HTA process. A proposition was made recently to combine the procedural ethics of the A4R with the substantive ethics set forth in the reflexive multicriteria framework EVIDEM (Evidence and Values: Impact on DEcision-Making).^11^ The framework postulates that criteria should evaluate whether the intervention appraised contributes to the goals the healthcare system, the triple aim,^12^ and whether it contributes positively to the organisation of the system of care and the socio-political context of its implementation.^13,14^ This comes from the consideration that the main objective of a public healthcare system should be to promote health and well-being of the whole population and ensure the common good. This is embedded in the concept of global value, which frames processes based on the goal of agencies, and for which patient and public engagement is paramount since the ultimate goal is indeed to serve best the whole population in need. The conceptualization behind EVIDEM is therefore fundamentally different than that of the EDP, since it builds on the multidimensional goal of healthcare and social services; in this, it relates on substantive ethics and guides the questions to be asked and reflected upon, and the knowledge that needs to be collected to provide fair and reasonable answers. This concept was then transformed into operationalizable criteria and a pragmatic and ethical process, building on multicriteria methodology and procedural ethics, with the latest proposition to combine reflexive multicriteria with A4R. The EDP defines the steps of the process, building on procedural ethics, but does not provide a guidance for what should be considered and why.

This proposition was recently applied 360 degrees for the development of patient centred socially responsible clinical practice guidelines on oral immunotherapy for the management of patients with food allergy.^15^ To better take the patient perspective into account, the EVIDEM framework was further developed to include three additional criteria regarding patient empowerment, alignment of the intervention with patient individual values, as well as the impact of the innovation on patient finances. A reflexive process on five dimensions – socio-political, populational, clinical, organizational and economical – was carried involving directly input from more than 40 stakeholders. The clinical practice guidelines includes recommendations not only regarding the delivery of care, but also on the critical importance of fostering a trusting relationship between the patient or caregiver and the clinical team, on innovative organisation of care to ensure quality of care and access, treatment choices that promotes sustainability as well as stewardship and development of healthcare system capacity to tailor to patient needs, and encourage care that promotes a positive evolution of the socio-political context surrounding food allergy towards a shared responsibility between patients and the healthcare system. This was made possible by: (1) the consideration of the multiple dimensions and the ethical issues they raise; (2) the involvement of patients and caregivers at each step, including during the deliberation leading to the recommendations; and (3) rich and deep reflexion from all participants on what, as a society we believe is the best for patients in need and the population as a whole.^15^ The process illustrates that, when geared towards social responsibility and patient centred, the Clinical practice guideline exercise can actually contribute to such goals; if circumscribed to the clinical aspect, the opportunity to contribute to the common good may be lost. The question is then, why should we limit the scope if there is an opportunity to contribute to more? We call for a reflexion on the value of such goal-oriented approaches, their limits in the current context and their potential contribution to inspiring the evolution of HTA towards patient centred and socially responsible recommendations for public financing and for clinical practice.

We concur with Culyer that a reflection is indeed needed to “enhance the fairness and legitimacy of HTA,” define “more clearly the authoritative scope of HTA” and “its effectiveness in addressing the issues of concern to decision-makers,” and the chief interested parties. Does the HTA process cover all relevant aspects, all relevant stakeholders? How can the agency make sure it does? Is the information made public helpful, necessary and sufficient for appropriation and pondering by all stakeholders? We agree with Culyer that HTA will not need a fundamental change but rather a refocus on what matters to make fair and reasonable recommendations. In addition, a reasoned prioritization of interventions on which to make recommendations will be needed for HTA to contribute as much as possible to the creation of global value for patients and population served.

Finally, we ponder on the concluding remark of Culyer on the need of ‘learning from doing what works best’ and that ‘In the absence of a theory of processes, we need to encourage imaginative innovation and much sharing of experience….(from) which some general principles might eventually be inferred.’ As a guidance for such endeavour, we could refer to the universal theory of process which articulates seven general steps that occurs between the potential of a process and the realization of its goal.^16^ This theory has some conceptual commonalities with the well-established principles of global quality systems and the reliance on plan-do-check-act improvement cycles.^17^ Both could inspire the development of core principles by which HTA agencies shall deliver their promises of promoting excellence, fair allocation of healthcare resources, for the benefit of the served populations. So, yes learning by doing, while keeping the goal in mind at each step of the process.

## Concluding Remarks

In a context of accelerating technicalization of healthcare, further research to understand how processes support the mission and values of HTA agencies appears needed to stimulate an international reflection on the what works best and what needs to be adjusted. Now is the time to fully seize the capacity of multidimensional approaches and of patient and public involvement to ensure that HTA is in sync with its environment, patient-centred, aimed at benefiting the whole population, and thus supports public healthcare systems in fulfilling their goals. This collective reflection could serve as a mean to synergize experiences and perspectives across the INHATA community and other institutions, to transform processes in a manner preparing us to tackle the ethical challenges of the 21st century, and ensure that HTA are focused on patients and population needs.^18^

## Acknowledgements

We would like to thank our collaborators from several regions of the world who share this vision and inspire us in preparing this piece.

## Ethical issues

Not applicable.

## Competing interests

Authors declare that they have no competing interests.

## Authors’ contributions

Both MG and MC developed the outlined for the publication, and drafted the text based on a common vision on the topic.

## Authors’ affiliations

^1^Department of management, Evaluation and Health Policy, School of Public Health, University of Montreal, Montreal, QC, Canada. ^2^Research Center, University Hospital Center Ste Justine, Montreal, QC, Canada.
